# Tracking False Lumen Remodeling with AI: A Variational Autoencoder Approach After Frozen Elephant Trunk Surgery

**DOI:** 10.3390/jpm15100486

**Published:** 2025-10-11

**Authors:** Anja Osswald, Sharaf-Eldin Shehada, Matthias Thielmann, Alan B. Lumsden, Payam Akhyari, Christof Karmonik

**Affiliations:** 1 Department of Thoracic and Cardiovascular Surgery, West-German Heart and Vascular Centre, University Duisburg-Essen, 45122 Essen, Germany; sharaf-eldin.shehada@uk-essen.de (S.-E.S.); matthias.thielmann@uk-essen.de (M.T.); payam.akhyari@uk-essen.de (P.A.); 2 Department of Vascular Surgery, Houston Methodist DeBakey Heart & Vascular Center, Houston, TX 77030, USA; 3 Translational Imaging Centre, Houston Methodist Research Institute, Houston, TX 77030, USA

**Keywords:** false lumen thrombosis, artificial intelligence algorithm, unsupervised learning, aortic dissection, aortic remodeling, personalized medicine, AI tools

## Abstract

**Objective:** False lumen (FL) thrombosis plays a key role in aortic remodeling after Frozen Elephant Trunk (FET) surgery, yet current imaging assessments are limited to categorical classifications. This study aimed to evaluate an unsupervised artificial intelligence (AI) algorithm based on a variational autoencoder (VAE) for automated, continuous quantification of FL thrombosis using serial computed tomography angiography (CTA). **Methods:** In this retrospective study, a VAE model was applied to axial CTA slices from 30 patients with aortic dissection who underwent FET surgery. The model encoded each image into a structured latent space, from which a continuous “thrombus score” was developed and derived to quantify the extent of FL thrombosis. Thrombus scores were compared between postoperative and follow-up scans to assess individual remodeling trajectories. **Results:** The VAE successfully encoded anatomical features of the false lumen into a structured latent space, enabling unsupervised classification of thrombus states. A continuous thrombus score was derived from this space, allowing slice-by-slice quantification of thrombus burden across the aorta. The algorithm demonstrated robust reconstruction accuracy and consistent separation of fully patent, partially thrombosed, and completely thrombosed lumen states without the need for manual annotation. Across the cohort, 50% of patients demonstrated an increase in thrombus score over time, 40% a decrease, and 10% remained unchanged. Despite these individual differences, no statistically significant change in overall thrombus burden was observed at the group level (*p* = 0.82), emphasizing the importance of individualized longitudinal assessment. **Conclusions:** The VAE-based method enables reproducible, annotation-free quantification of FL thrombosis and captures patient-specific remodeling patterns. This approach may enhance post-FET surveillance and supports the integration of AI-driven tools into personalized aortic imaging workflows.

## 1. Introduction

Artificial intelligence (AI) methods are increasingly utilized to enhance imaging-based assessments in cardiovascular surgery, facilitating advanced image analysis, precise risk prediction, and personalized treatment planning. In the field of aortic surgery specifically, AI-driven tools have shown promise in tasks such as automated measurement of aortic dimensions, detection of endoleaks, and prediction of clinical outcomes following dissection or aneurysm repair [1,2]. However, many existing AI approaches rely on supervised learning techniques, which require extensive, manually annotated datasets—datasets that are challenging to obtain for rare or anatomically complex conditions such as aortic dissection. Unsupervised learning approaches, particularly variational autoencoders (VAEs), provide a powerful alternative by autonomously extracting latent imaging features. VAEs encode images into structured latent representations, enabling scalable, consistent, and robust analyses across diverse clinical data [3,4,5].

The Frozen Elephant Trunk (FET) procedure offers a single-stage solution for complex aortic pathologies by combining surgical replacement of the aortic arch with endovascular stent grafting of the descending aorta [6,7]. In aortic dissections, FET promotes blood flow redirection into the true lumen (TL) while excluding the false lumen (FL) [8]. This promotes FL thrombosis, a critical step toward favorable aortic remodeling, characterized by FL shrinkage and/or TL enlargement [9]. Complete or partial thrombosis of the FL has been associated with a reduced risk of aneurysmal degeneration and rupture, while a persistent patent FL correlates with increased reinterventions and a poorer long-term prognosis [10,11].

Given its clinical importance, postoperative monitoring of FL thrombosis status via serial computed tomography angiography (CTA) is crucial. Currently, however, FL assessment primarily relies on manual image interpretation, a time-consuming, subjective process susceptible to inter-observer variability.

Furthermore, conventional assessments typically categorize the FL into simple classes such as patent, partially thrombosed, or completely thrombosed, offering limited quantitative granularity for precise tracking of disease progression.

To address these challenges, we recently introduced an artificial intelligence (AI)-based, unsupervised classification algorithm using a 2D convolutional neural network (CNN) embedded within a variational autoencoder (VAE) framework [12]. Trained on centerline-based cross-sectional aortic images, this approach automatically identifies and quantifies FL thrombus without manual annotations. Our initial feasibility study on a small patient cohort demonstrated high agreement between the algorithm’s assessments and expert evaluations, introducing a quantitative, patient-specific “thrombus score” that captures the extent and distribution of FL thrombosis. VAEs offer several advantages particularly suited to medical image analysis. Their structured latent space enables them to capture subtle anatomical and pathological variations, such as transitions among patent, partially thrombosed, and fully thrombosed FL segments [13].

Building on our earlier feasibility study, the present work applies the VAE-based thrombus classifier to a larger, more diverse cohort of patients who underwent FET surgery. By analyzing paired postoperative and follow-up CTAs from thirty patients, we aim to evaluate the evolution of FL thrombosis over time and assess the potential of AI-assisted thrombus quantification to enhance postoperative surveillance and clinical decision-making.

## 2. Materials and Methods

### 2.1. Patient Cohort and Imaging Data

This retrospective study was approved by the institutional ethics committee of the University of Duisburg-Essen, Essen, Germany (23-11102-BO). Due to its retrospective design, the requirement for individual patient consent was waived. CTA datasets were collected from a total of 30 patients who underwent the FET procedure for acute or chronic aortic dissection between 08/2017 and 07/2019. Of those, 20 patients presented with acute aortic dissection and 10 with chronic aortic dissection. From the institutional database, patients were randomly selected under the criterion that both a postoperative CTA and at least one follow-up CTA were available, enabling longitudinal analysis. The mean follow-up interval was 4.05 ± 2.12 years.

FL status was determined based on contrast enhancement in the CTA images and categorized as obliterated, fully thrombosed, partially thrombosed, or patent.

### 2.2. Image Reconstruction and Preprocessing

Segmentations of the true lumen, false lumen, and thrombus were performed in Osirix (Pixemo Sarl, Bernex, Switzerland, version 14.0). To standardize analysis across patients and timepoints, a centerline through the aortic lumen was generated using the 3D curved multiplanar reconstruction tool. This centerline allowed for reconstruction of cross-sectional images perpendicular to the aortic lumen, thereby centering the vessel within each slice and minimizing variation in orientation. Segmented images were then automatically cropped and centered, with a final image resolution of 64 × 64 pixels. This standardized image format was used as input for the variational autoencoder (VAE) model.

### 2.3. Variational Autoencoder Architecture

The artificial intelligence model was based on a VAE, a type of unsupervised neural network capable of encoding input data into a structured latent space and reconstructing the original image from that compressed representation. Both the encoder and decoder consisted of convolutional neural networks, specifically designed to process 2D medical images.

The encoder CNN reduced the dimensionality of input images and mapped them to a two-dimensional latent space. The decoder CNN then reconstructed images from these latent variables. The VAE was implemented using TensorFlow (version 2.7.0) in Python (version 3.9.9) within a Conda environment (version 4.11.0). The total number of images to train the VAE were 16,181. Model training was performed over 100 epochs to ensure convergence and optimal reconstruction quality.

### 2.4. Latent Space Analysis and Image Classification

**Figure 1:**
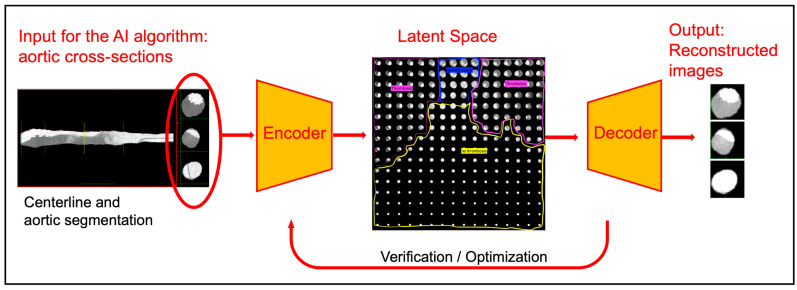
Workflow of the variational autoencoder–based analysis. Aortic cross-sections, derived from centerline reconstruction and segmentation, serve as input to the algorithm. The encoder projects each slice into a two-dimensional latent space, where regions corresponding to no thrombus (yellow), partially patent false lumen (blue), and thrombosed false lumen (purple) can be distinguished. The decoder reconstructs the images, allowing verification of encoding accuracy. The radial distance in the latent space is used as a continuous thrombus score, enabling quantitative assessment of false lumen remodeling.

To separate images with and without thrombus, a boundary using parameterized polygon functions was established in the latent space based on the distribution of encoded images. After initial manual selection of boundaries, polygon parameterization using the Polygon () function in Python, to define areas in the 2D latent space to designate regions with (a) no thrombus, (b) partially patent false lumen and (c) thrombosed false lumen (Figure 1).

**Figure 2:**
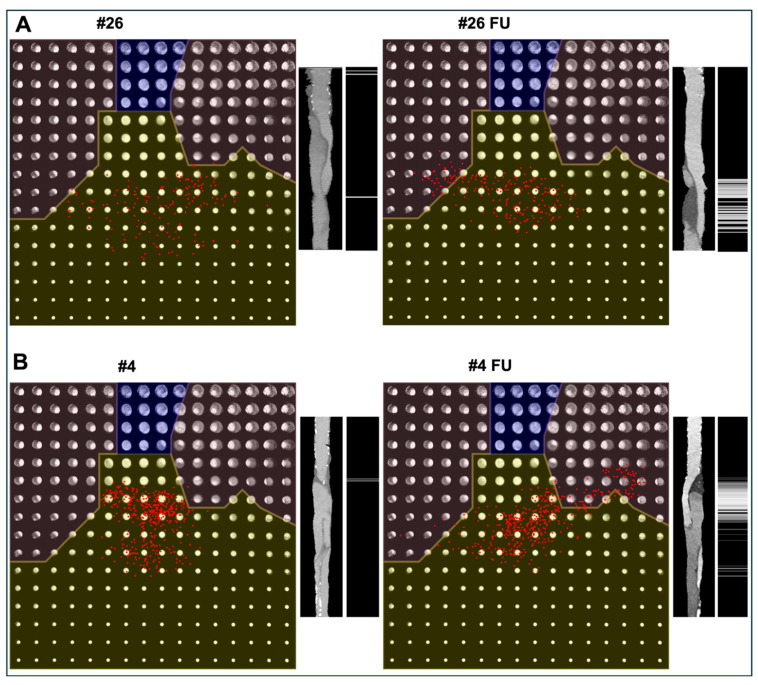
Representative examples of thrombus progression ((**A**), Patient #26) and regression ((**B**), Patient #4) after FET surgery (left = postoperative, right = follow-up). On the left side of each panel, the two-dimensional latent space generated by the variational autoencoder is shown. The latent space is divided into three clinically interpretable regions: no thrombus (yellow), partially thrombosed false lumen (unshaded), and fully thrombosed false lumen (blue). Each red dot represents one axial aortic slice encoded into the latent space. The radial distance from the origin corresponds to the thrombus score, with larger distances indicating greater false lumen thrombus burden. On the right side of each panel, a multiplanar reconstructed lateral view of the aorta along the centerline is displayed, and a grayscale map illustrates the slice-wise thrombus score (black = no thrombus; white = maximum thrombus burden).

Thrombus score was calculated as follows for each slice: zero for regions with no thrombus, sqrt(x^2 + y^2) for regions with thrombus accounting for progressively larger thrombus burden with increasing distance from the center of latent space and 1/3*sqrt(x^2 + y^2) to partially patent lumen. The total thrombus score for each case was obtained by summing up the thrombus score values for all slices and divided by the number of slices (File S1). This averaging step ensured that the score was independent of scan length or slice thickness and therefore comparable between patients. Finally, all thrombus scores were normalized to the range [0,1] to enable comparability across patients and between postoperative and follow-up scans (Figure S1). Baseline and follow-up latent spaces for two patients are shown in Figure 2A,B together with a representative lateral cross-section obtained for a multiplanar reconstruction along the aortic centerline and corresponding thrombus score for each slice. For patient one, the increase in thrombus score is reflective of false lumen thrombosis. For patient two, the presence of thrombus decreased with corresponding disappearance of thrombotic region.

For each patient dataset, an average thrombus score was computed by aggregating the scores of all individual slices.

### 2.5. Visualization and Validation

For qualitative validation, each axial image was mapped back from the latent space using the decoder and compared visually with the original input to ensure fidelity of reconstruction. Additionally, thrombus score distributions were visualized along the length of the aorta using grayscale lateral representations along curved MPR lateral image cross-sections, allowing for anatomical correlation.

### 2.6. Statistical Analysis

To assess whether the thrombus scores differed significantly between baseline and follow-up timepoints, first the distribution of the paired score differences for normality using the D’Agostino and Pearson omnibus test was evaluated. A paired *t*-test was then applied to determine whether the mean difference between paired observations differed significantly from zero. A two-sided *p*-value < 0.05 was considered statistically significant. Statistical analyses were performed using Python’s SciPy library.

## 3. Results

**Figure 3:**
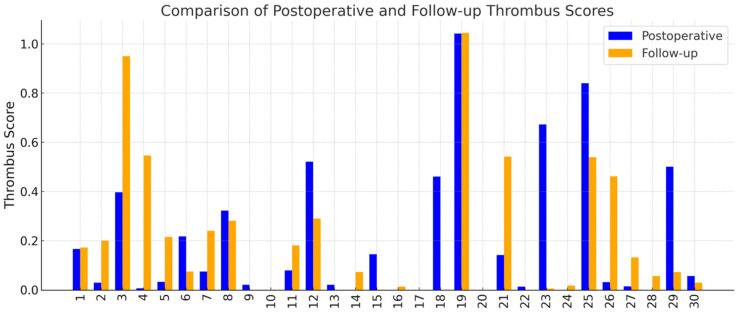
Patient-specific thrombus scores derived from cross-sectional CTA images using the VAE model. Each pair of bars represents one patient, with the blue bar showing the score at the early postoperative scan and the orange bar showing the score at follow-up. The *y*-axis shows the average thrombus score.

The VAE-based classifier successfully computed thrombus scores for all 30 patients based on cross-sectional CT images at two timepoints: postoperative and follow-up. Thrombus scores were derived slice-wise, aggregated per patient, and used to quantitatively characterize the extent of FL thrombosis. Individual thrombus scores at each timepoint are illustrated in Figure 3, highlighting considerable inter-patient variability in thrombus burden and its longitudinal development.

Among the cohort, 15 patients (50.0%) demonstrated an increase in thrombus score over time, suggesting favorable aortic remodeling. In contrast, 12 patients (40.0%) demonstrated a decrease in thrombus score, which may indicate regression of the thrombosed false lumen. The remaining 3 patients (10.0%) showed no change in their thrombus score between baseline and follow-up assessments (Table 1).

**Figure 4:**
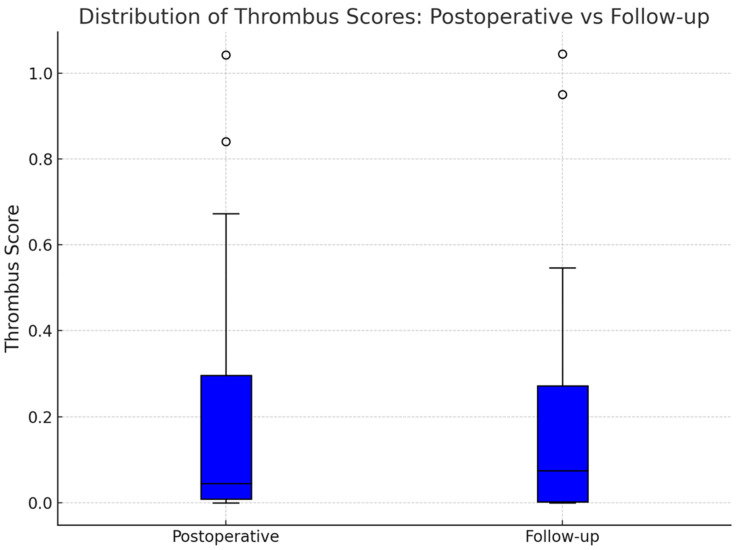
This boxplot compares the distribution of thrombus scores across the entire cohort at the two imaging timepoints. The median thrombus score increased slightly underscoring the slight trend towards increased false lumen thrombosis. Outliers at both timepoints reflected patients with near-complete FL thrombosis or persistent FL patency, demonstrating the spectrum of clinical presentations within the cohort.

**Figure 5:**
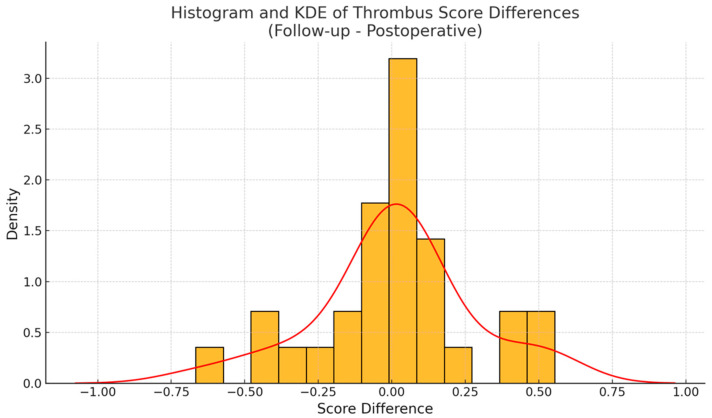
The histogram of thrombus score differences (yellow) shows a skew toward positive values. The smoothed kernel density estimated curve (red line) peaked just above zero, suggesting that a modest majority of patients experienced thrombus gain over time.

Although individual remodeling trajectories were diverse, a modest trend toward Okincreased FL thrombosis was observed at the population level. Boxplots of thrombus scores showed a slight upward shift in the distribution at follow-up (Figure 4). Additionally, a histogram of thrombus score differences (Figure 5) revealed a right-skewed distribution, with a smoothed kernel density estimate curve that peaked just above zero, further supporting the notion of mild FL thrombosis progression in a subset of patients.

To formally assess changes in thrombus scores over time, we first evaluated the distribution of paired differences for normality. The D’Agostino and Pearson omnibus test did not reveal any significant deviation from a normal distribution (statistic = 1.79, *p* = 0.41), validating the use of a paired *t*-test for further analysis. The paired *t*-test comparing thrombus scores at baseline and follow-up yielded a t-statistic of 0.23 and a two-sided *p*-value of 0.82, indicating no statistically significant difference in mean thrombus scores across the full cohort. These findings imply that, while many patients experienced relevant individual changes in FL thrombus burden, these shifts were bidirectional and canceled each other out at the group level.

In addition to the continuous thrombus score analysis, a categorical assessment of false lumen status was performed according to standard clinical evaluation and is summarized in Table 2. In the stent-graft covered thoracic aorta, 26 patients showed complete thrombosis postoperatively, while at follow-up 16 patients demonstrated obliteration and 10 remained thrombosed. Distal segments were predominantly patent after surgery (23/30 from DLZ to coeliac trunk, 26/30 from coeliac trunk to aortic bifurcation) and continued to show high rates of patency at follow-up, although cases of partial thrombosis and obliteration were also observed.

## 4. Discussion

The presence and evolution of FL thrombosis following FET surgery are critical determinants of aortic remodeling and long-term patient outcomes. Complete or progressive FL thrombosis is generally associated with favorable remodeling, reduced rates of reintervention, and improved survival, whereas persistent FL patency correlates with adverse remodeling and an elevated risk of late complications [9,14,15]. However, traditional methods typically categorize the FL status as patent, partially thrombosed, fully thrombosed or obliterated and are therefore inherently limited due to the lack of quantitative granularity. A more nuanced and quantitative assessment is often reserved for research applications and has not yet translated into routine clinical workflows. To address this gap, we introduce a continuous, image-derived “thrombus score” based on an unsupervised deep learning model to enable objective, individualized tracking of FL thrombosis over time.

AI methods, particularly deep learning algorithms, have shown increasing promise in enhancing reproducibility and objectivity in cardiovascular imaging. Recent developments using CNNs and VAEs have enabled automated segmentation, feature extraction, and even outcome prediction in aortic diseases. While supervised learning methods have demonstrated high performance in tasks such as thrombus detection, they require large, high-quality annotated datasets that are difficult to obtain in rare and anatomically complex conditions like aortic dissection [16]. In contrast, unsupervised approaches like VAEs can capture latent morphological patterns directly from raw imaging data without manual labeling, making them more scalable and adaptable to diverse clinical scenarios.

VAEs have shown robust performance in modeling large-scale anatomical structures in various medical domains. For example, they have been successfully applied to myocardial segmentation and cardiac remodeling, as well as in the quantification of vascular lesions in peripheral arterial disease [17,18]. Their ability to encode complex anatomical variation with minimal supervision makes them particularly well-suited to assess thrombus morphology, which typically presents as spatially distinct, low-attenuation regions in CTAs. Our findings further support this strength, demonstrating that VAEs can be leveraged to extract clinically relevant features in a reproducible, data-efficient manner. In addition to algorithmic advances, the clinical translation of AI methods depends critically on usability, interpretability, and integration into established workflows. A recent study demonstrated that complex image analysis techniques can be transformed into clinician-friendly pipelines using accessible tools and minimal programming expertise [19]. Such usability considerations are essential for translation: an AI method must not only perform well technically, but also be interpretable, workflow-compatible, and practical for clinicians.

In this study, we applied a VAE-based unsupervised AI algorithm to quantify and longitudinally track FL thrombosis in a cohort of 30 patients who underwent FET surgery, each with baseline and follow-up CTA scans. The model, which had previously demonstrated strong reconstruction fidelity in a feasibility setting, was now evaluated in a clinically relevant longitudinal context [12]. The heterogeneous remodeling patterns observed—thrombus progression in 50% of patients, regression in 40%, and stability in 10%—highlight the individualized nature of post-FET aortic healing. The variation in the magnitude of thrombus score change further reflects patient-specific factors such as anatomical variability, surgical technique, and hemodynamic conditions [20]. A categorical assessment of FL status, summarized in Table 2, confirmed stable thrombosis in the stent-graft covered thoracic aorta and divergent courses in distal segments, ranging from persistent patency to complete obliteration. The lack of a significant group-level change (*p* = 0.82) further emphasizes the heterogeneity of remodeling. Subgroup analyses may provide additional insight, particularly when differentiating between acute and chronic dissections. Although our cohort was too small to allow formal stratification, these findings underscore the potential of quantitative, patient-specific metrics over categorical assessments to better capture the complexity of aortic remodeling.

A recent study by Zhuang et al. introduced a fully automated pipeline for the identification, segmentation, and Stanford subtyping of aortic dissection from CTA, integrating aorta and TL/FL segmentation with multi-view classification [21]. Similar to our approach, their work demonstrates the potential of deep learning algorithms to streamline image analysis and reduce the need for manual annotations, thereby improving reproducibility and scalability in the assessment of aortic disease. Both methods therefore share the overarching goal of leveraging AI to enable objective and standardized evaluation of complex aortic pathologies. The main distinction lies in the clinical application: while Zhuang et al. focused on diagnostic classification and subtype differentiation, our study applies unsupervised learning to the longitudinal quantification of false lumen thrombosis after FET surgery. By introducing a continuous VAE-derived thrombus score, our method extends AI-based imaging beyond categorical classification toward individualized postoperative surveillance and monitoring of remodeling dynamics.

The longitudinal application of this AI-based method offers significant clinical value. Serial imaging provides critical insight into disease dynamics and therapeutic response. The thrombus score, derived automatically and reproducibly, could serve as an objective marker of remodeling trajectory—informing individualized follow-up intervals, therapeutic efficacy, and timing of secondary interventions.

In addition to anatomical variability, surgical technique, and local hemodynamic conditions, systemic coagulation factors may also influence the course of FL thrombosis and remodeling. Patients with an underlying prothrombotic disposition, such as thrombophilia or antiphospholipid syndrome, might experience different remodeling trajectories even in the absence of a prior diagnosis. Since the process of FL thrombosis is central to favorable remodeling, future prospective studies should explore potential correlations between individual coagulation profiles and imaging-based markers of remodeling. Integrating such systemic factors with AI-derived imaging parameters could provide a more comprehensive understanding of patient-specific outcomes after FET surgery.

Moreover, in the context of peripheral arterial disease, the algorithm’s performance was extensively tested with a Gaussian mixture model and achieved classification probabilities between 0.82 and 1.00 for hard tissue-containing lesions, which were defined by distinct structural features such as calcification and occlusion [22]. In contrast, the same algorithm showed lower and more variable classification probabilities (0.56–0.93) for soft-tissue-rich lesions, where the boundaries and internal patterns were more complex and less distinct.

This finding supports a key strength of VAEs: their ability to encode well-delineated anatomical features with high reliability. Given that aortic thrombus typically presents as a large, spatially distinct filling defect on CT imaging, this characteristic makes it well-suited for analysis using VAE-based classification. In our application, the algorithm demonstrated consistent performance in capturing thrombus presence and progression, leveraging these large and easily separable features in the latent space to deliver reproducible thrombus quantification.

### Limitations

The retrospective design prevented the standardization of imaging protocols, and variations in contrast timing, slice alignment, and scanner differences may have influenced image quality and model output. Such variability in image acquisition can obscure subtle thrombus changes over time and may also introduce noise into the latent space representation, thereby affecting the stability of the thrombus score. Moreover, calculating average thrombus scores across the entire aorta may mask segment-specific remodeling behaviors, as the FL remodeling differs considerably between the aortic segments [9]. In addition, clinical parameters such as coagulation disorders were not systematically assessed, and clinical endpoints including reintervention, mortality, or complications were not evaluated. As a result, potential systemic influences on FL thrombosis and the prognostic value of the thrombus score remain uncertain. The relatively small sample size limits the statistical power and generalizability of the findings, underscoring the need for larger validation cohorts. Finally, although the mean follow-up period of 4.05 ± 2.12 years appears sufficient to capture relevant remodeling at the stented thoracic level, the aorta undergoes continuous long-term change. Future studies with standardized imaging, larger cohorts, and multiple evaluation timepoints (e.g., postoperatively, 1 year and at last follow-up) will be required to validate the robustness and clinical utility of this method.

## 5. Conclusions

This study demonstrates the feasibility and potential clinical value of an unsupervised VAE model for the automated, quantitative assessment of FL thrombosis in patients after FET surgery. The introduction of a continuous, image-derived thrombus score enabled tracking of individualized remodeling patterns over time, which are not captured by conventional categorical classifications. The lack of a significant group-wide change, together with marked variation between patients, highlights the heterogeneity of post-dissection remodeling and the importance of individualized monitoring.

Unsupervised AI approaches such as the VAE classifier can provide reproducible, annotation-free quantification of imaging features and may complement standard radiological assessment. This could improve risk stratification and support personalized follow-up strategies after FET. Larger studies with clinical outcome data are needed to confirm these findings and determine their prognostic value.

## Figures and Tables

**Table 1:** Descriptive statistics of thrombus score in the patient population.

Change	Number of Patients	Mean Difference	Min. Difference	Max. Difference
Increased	15	0.189	0.002	0.554
Unchanged	3	0.000	0.000	0.000
Decreased	12	–0.208	–0.667	–0.014

**Table 2:** False lumen status postoperatively and at follow-up according to standard clinical assessment, stratified by aortic segment (stent graft (SG) covered thoracic aorta, distal landing zone (DLZ) to coeliac trunk, and coeliac trunk to aortic bifurcation).

False Lumen Status	Postoperative (*n* = 30)			Follow-Up (*n* = 30)		
	SG Area	DLZ to Coeliac Trunk	Coeliac Trunk to Aortic Bifurcation	SG Area	DLZ to Coeliac Trunk	Coeliac Trunk to Aortic Bifurcation
obliterated	0	4	4	16	8	4
thrombosed	26	0	0	10	2	0
partial	4	3	0	3	6	0
patent	0	23	26	1	14	26

## Data Availability

The raw data supporting the conclusions of this article will be made available by the authors on request.

## Supplementary Materials

The following supporting information can be downloaded at: https://www.mdpi.com/article/10.3390/jpm15100486/s1, File S1: Thrombus Score Calculation; Figure S1: Schematic illustration of the latent space and thrombus score. The two-dimensional latent space generated by the variational autoencoder is shown, with regions corresponding to no thrombus (yellow), partially thrombosed false lumen (unshaded), and fully thrombosed false lumen (blue). Each red dot represents an axial slice projected into the latent space, with the origin at the center. The radial distance from the origin reflects the thrombus score, with larger distances indicating greater thrombus burden (arrows). On the right, a multiplanar reconstructed aortic centerline and the corresponding slice-wise grayscale map are shown (black = no thrombus, white = maximum thrombus burden). The patient-level thrombus score in this example is 0.68.

## Abbreviations

AI	artificial intelligence
CNN	convolutional neural networks
CTA	computed tomography angiography
FET	Frozen Elephant Trunk
FL	false lumen
TL	true lumen
VAE	variational autoencoder
