# Supplementary material for: Tracking False Lumen Remodeling with AI: A Variational Autoencoder Approach After Frozen Elephant Trunk Surgery
Source: J Pers Med. 2025 Oct 11;15(10):486. doi: 10.3390/jpm15100486 (PMC12565649; doi:10.3390/jpm15100486)
Supplement: Supplementary file 1 [file jpm-15-00486-s001.zip › jpm-3866206-supplementary.pdf]

File S1: Thrombus Score Calculation

For each axial slice, the thrombus score was defined as follows:

$$S_{slice} = \begin{cases} 0, & if\ classified\ as\ no\ thrombus \\ \sqrt{x^2 + y^2}, & if\ classified\ as\ thrombosed \\ \frac{1}{3}\sqrt{x^2 + y^2}, & if\ classified\ as\ partially\ patent \end{cases}$$

where (x, y) are the latent-space coordinates normalized between 0 and 1.

The total thrombus score for each patient was calculated as the mean of all slice-level scores:

$$S_{patient} = \frac{1}{N} * \sum_{i=1}^{N} S_{Slice,i}$$

where N is the total number of slices. This procedure ensures that patient-level scores are independent of the number of slices and thus comparable across scans and patients.

Figure S1:

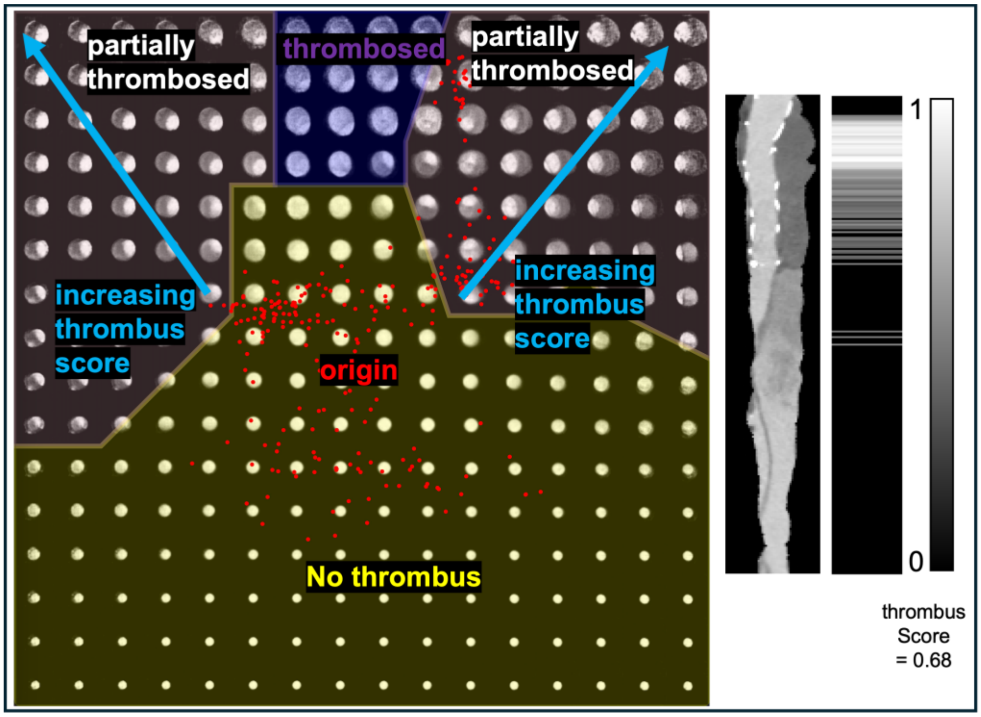

Figure S1. Schematic illustration of the latent space and thrombus score. The two-dimensional latent space generated by the variational autoencoder is shown, with regions corresponding to no thrombus (yellow), partially thrombosed false lumen (unshaded), and fully thrombosed false

lumen (blue). Each red dot represents an axial slice projected into the latent space, with the origin at the center. The radial distance from the origin reflects the thrombus score, with larger distances indicating greater thrombus burden (arrows). On the right, a multiplanar reconstructed aortic centerline and the corresponding slice-wise grayscale map are shown (black = no thrombus, white = maximum thrombus burden). The patient-level thrombus score in this example is 0.68.
